# Autophagy-Regulating, Photothermal Polydopamine-Coated, and Photodynamic Zirconium/Porphyrin-Framed Metal–Organic Frameworks for Enhanced Doxorubicin Therapy in Colon Cancer

**DOI:** 10.34133/bmr.0218

**Published:** 2025-06-12

**Authors:** Junghan Lee, Kwangsun Yu, Enkhzaya Davaa, Ratchapol Jenjob, Phuong Hoa Tran, Dahee Ryu, Jongyoon Kim, Seongju Lee, Zheyu Shen, Wha-Seung Ahn, Chung-Sung Lee, Su-Geun Yang

**Affiliations:** ^1^ Department of Biomedical Science, BK21 FOUR Program in Biomedical Science and Engineering, Inha University College of Medicine, Incheon 22332, Republic of Korea.; ^2^ Carbon Neutral Demo-Plant Center, Korea Research Institute of Chemical Technology (KRICT), Yeosu-si59631, Republic of Korea.; ^3^ Department of Chemical Engineering, Inha University, Incheon 22201, Republic of Korea.; ^4^ School of Biomedical Engineering, Southern Medical University, Guangzhou, Guangdong 510515, China.; ^5^ Department of Pharmaceutical Engineering, Soonchunhyang University, Chungcheongnam-do 31538, Republic of Korea.

## Abstract

Metal–organic frameworks (MOFs) have immense potential for biomedical applications. This paper reports the development of multifunctional zirconium-based metal–organic framework (ZrMOF) nanohybrids, featuring a photodynamic porphyrin-framed zirconium cluster with photothermal polydopamine (PD) coating. The PD-coated ZrMOF (PD/ZrMOF) nanohybrids exhibit enhanced colloidal stability and biocompatibility. The PD/ZrMOF nanohybrids in the present study exhibited a unique combination of functionalities, including photodynamic therapy (PDT), photothermal therapy (PTT), and the delivery of anticancer agents. Furthermore, hydrazone-modified doxorubicin (DOX-hyd) was encapsulated within the PD/ZrMOF nanohybrids, enabling a pH-responsive release mechanism that responds to acidic conditions within the tumor microenvironment. This study examined how MOFs influence autophagy, which is essential for maintaining cellular homeostasis in various human diseases, resulting in autophagy activation by MOF treatment. Additional research into the possible mechanisms of autophagy by MOF showed that the up-regulation of Beclin-1 and ATG7, independent of the mTOR pathway, contributes to autophagy induction. Furthermore, the DOX-hyd-encapsulated PD/ZrMOF nanohybrids (DOX-hyd-PD/ZrMOF) exhibited remarkable cancer suppression ability in vitro and in vivo, owing to their tri-mode therapeutic capabilities comprising PDT, PTT, and chemotherapy. This versatile “three-in-one” nanoplatform enables efficient cancer imaging and offers a powerful strategy for multi-mode combination treatments.

## Introduction

Metal–organic frameworks (MOFs) are a class of highly ordered crystalline nanoscale materials assembled by metal ions/clusters and organic ligands [1,2]. MOFs with various intriguing properties, including high surface area, porosity, adjustable composition, and biocompatibility, have attracted considerable attention as promising candidates for biomedical applications such as sensing, medicine, and imaging [3–5]. Various pharmaceutical agents, such as anticancer drugs, imaging probes, and genetic molecules, can be incorporated into MOFs with specific nanostructures [6]. Especially, zirconium-based MOFs (ZrMOFs) have shown promising potential in cancer therapy due to their structural tunability and versatility. For example, drug delivery systems based on ZrMOFs have attracted increasing attention because of their excellent biocompatibility, low toxicity, and high affinity to the phosphate groups of biomolecules [7–9]. ZrMOFs, synthesized with porphyrinic photosensitizers as an organic building block, displayed an application potentials for photodynamic therapy (PDT), fluorescence imaging, and drug delivery in cancer treatments [10,11]. In addition, ZrMOFs have been investigated for photothermal cancer ablation with low side effects, resulting from inducing apoptosis in cancer cells through spatiotemporal hyperthermia (~50 °C) in response to specific wavelength light irradiation [12]. Although ZrMOFs have great potential for biological applications, their resistance and stability to moisture and physiological buffers are poorly understood. Furthermore, their biosafety evaluation has not been sufficiently studied [13]. The effects of PDT and photothermal therapy (PTT) as a single therapeutic option are limited by the insufficient generation of reactive oxygen species (ROS) and photothermal effect, resulting from the undesirable conversion efficiency of photoreactive agents because of the restricted tissue penetration depth of the light [14,15]. These studies suggest that further amendments are needed to achieve optimal colloidal stability of ZrMOFs in the conditioned medium. Therefore, more advanced design of versatile ZrMOFs, including multimodalities within a single system, to achieve the desired stability, therapeutic functions, is expected to help ensure effective cancer treatment.

Combining PDT, PTT, and chemotherapy to achieve an “all-in-one” modality offers various advantages for efficient cancer treatment [16]. In particular, the local heating induced by PTT enhances blood flow and oxygen supplementation, improving the local therapeutic efficiency of the PDT treatment. Furthermore, additional PDT treatment with PTT can enhance the sensitivity of cancer tissues, altering the physiological microenvironment of tumors. Therefore, ZrMOFs, as a facile and multimodal photoreactive nanosystem with the synergistic efficacy of PDT and PTT, can be a great cancer therapeutic candidate for achieving excellent cancer treatment.

Recent studies have shown that various nanomaterials can regulate autophagy and hold promise for applications in autophagy-related cancer therapies [17]. Verifying the relationship between autophagy and the functionalized ZrMOFs may offer a clearer understanding of their therapeutic mechanisms in cancer treatment. This study investigates how ZrMOF and surface-modified ZrMOFs (PD/ZrMOF and DOX-hyd-PD/ZrMOF) influence autophagy regulation, as well as their therapeutic effectiveness and overall potential in cancer therapy.

**Fig. 1.**
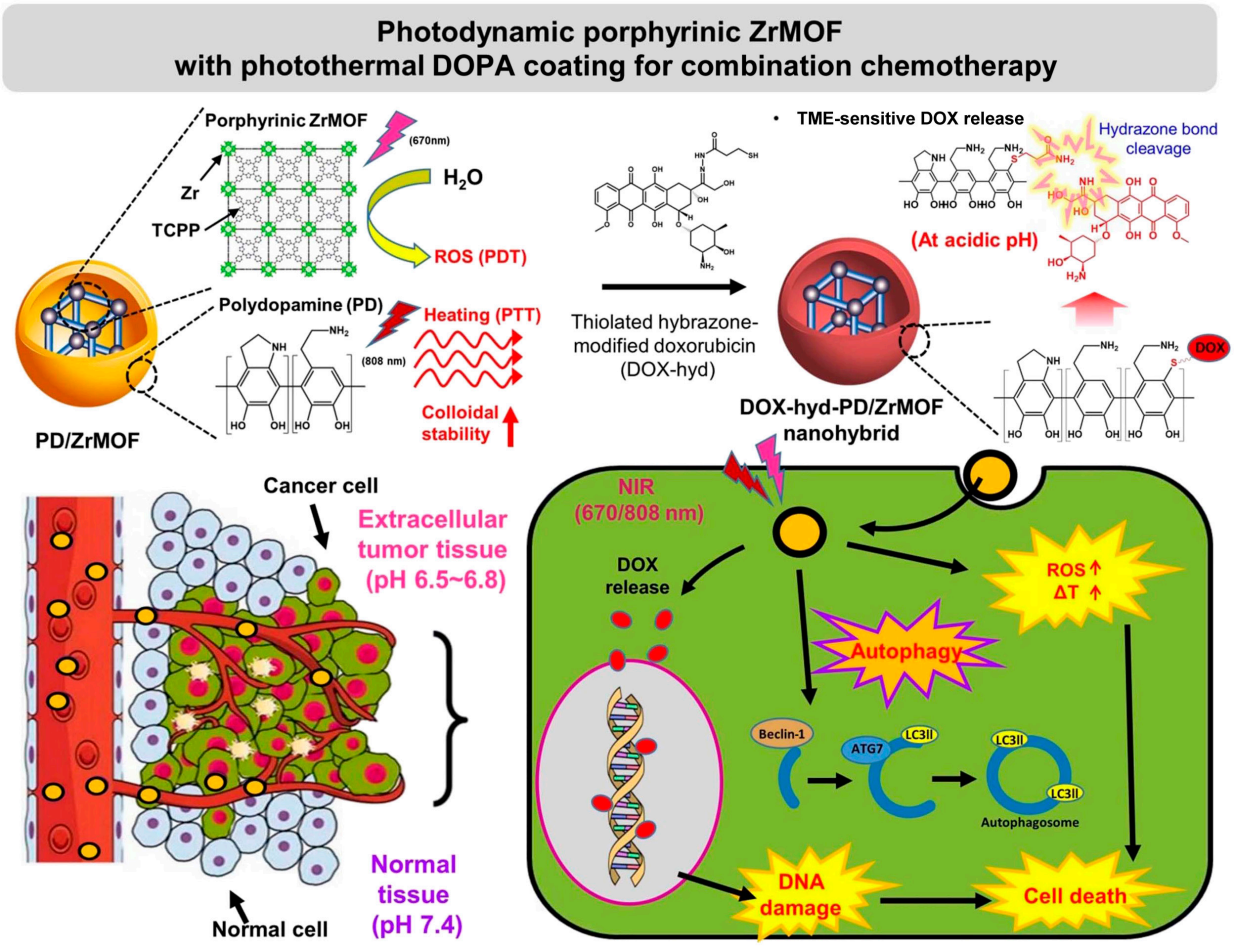
Design of multimodal Zr-based porphyrinic MOF nanohybrids stabilized with PD for photodynamic, photothermal, and pH-responsive chemotherapeutic effects.

Multifunctional ZrMOF nanohybrids were designed and developed with porphyrin ligand and Zr clusters, which were further stabilized with polydopamine (PD) via an oxidative self-polymerization assembly for multimodal colorectal cancer treatment (Fig. 1). Moreover, incorporating PD with a broad absorption range can increase the photothermal conversion efficiency by up to 40%, maximizing the PTT effect [18]. After coating the PD onto the ZrMOF, DOX-hyd was further assembled onto PD/ZrMOF nanohybrids via Schiff base formation and Michael-type addition between the PD and the primary thiol of the PD-covered ZrMOF nanohybrid (PD/ZrMOF) surface to enable its chemotherapeutic functionality [19].

This study evaluated various physicochemical properties, including singlet oxygen generation, photothermal response, colloidal stability, and the release properties of DOX in tumor microenvironments of the synthesized ZrMOF, PD/ZrMOF, and DOX-hyd-loaded PD/ZrMOF (DOX-hyd-PD/ZrMOF) nanohybrids. In addition, this study investigated the tri-modal properties of PDT, PTT, and chemotherapy of DOX delivery using a single nanohybrid system to achieve a substantial cancer suppression effect in both in vitro and in vivo systems and evaluated their potential therapeutic efficacy.

## Materials and Methods

### Materials

Zirconyl chloride octahydrate (ZrOCl_2_·8H_2_O; Sigma-Aldrich, USA, 98%), tetrakis (4-carboxyphenyl)porphyrin (H_2_TCPP; TCI, Japan, >97%), benzoic acid (C_7_H_6_O_2_; TCI, Japan, >99%), *N,N*-dimethylformamide anhydrous (C_3_H_7_NO; Sigma-Aldrich, USA, 99.8%), and acetone (C_3_H_6_O; Sigma-Aldrich, USA, 99.9%) were used as received.

### Synthesis of PD/ZrMOF nanohybrids

The modified synthetic method was conducted to synthesize smaller ZrMOF particles based on MOF-525 reported elsewhere [20]. Zirconyl chloride octahydrate (0.11 g) and benzoic acid (1.35 g) as a modulator were transferred to a vial with dimethylformamide (DMF, 32 ml), and the mixture solution was sonicated and heated in a conventional oven at 100 °C for 1 h. Subsequently, H_2_TCPP (0.05 g) was added to the cooled mixture solution and sonicated until complete dissolution. ZrMOFs were synthesized by heating the mixture solution in a conventional oven at 80 °C for 24 h. After cooling to room temperature, the resulting purple solid was filtered and washed 3 times using fresh DMF and acetone sequentially. The final product was obtained by drying in a vacuum chamber to remove the solvent from the pores of the ZrMOF. The PD/ZrMOF nanohybrids were prepared by adding dopamine (DOPA) hydrochloride (60, 90, and 120 mg) to 30 mg of ZrMOFs in a Tris buffer solution (pH 8.5) and stirred overnight at room temperature. The resulting PD/ZrMOF nanohybrids were washed 3 times with distilled water.

### Synthesis of DOX-hyd and DOX-hyd-PD/ZrMOF nanohybrids

DOX-hyd was synthesized by dissolving 200 mg of DOX hydrochloride and 120 mg of 3,3′-dithiobis(propanoic dihydrazide) in 10 ml of methanol solution containing 200 μl of trifluoroacetic acid (TFA), followed by thorough mixing. The solution was stirred at room temperature for 72 h in the dark, and the solvent was removed by rotary evaporation. The sample was washed 3 times with acetonitrile and lyophilized. The dried sample (150 mg) was redispersed in 10 ml of methanol. Subsequently, 100 mg of 1,4-dithiothreitol (DTT), a reducing agent, was added to activate the thiol group. After stirring for 24 h at room temperature, the final product (pH-cleavable DOX-hyd) was purified using a Sephadex G-10 column and freeze-dried. The drug was conjugated to PD/ZrMOF nanohybrids by mixing 150 mg of PD/ZrMOF nanohybrids with 75 mg of DOX-hyd in 5 ml of Tris buffer (pH 8.5) and stirred overnight at room temperature.

### Physicochemical analysis of the synthesized nanohybrids

The particle size and morphology of synthesized nanohybrids were measured using transmission electron microscopy (TEM; JEOL JEM-1011, JEOL, Tokyo, Japan) and a dynamic light scattering (DLS) (Zetasizer Nano ZS90, Malvern, UK). The x-ray diffraction (XRD; Rigaku) patterns were measured using CuKα radiation (λ = 1.5418 Ǻ).

### Singlet oxygen (^1^O_2_) generation of nanohybrids

The generation of ROS by the nanohybrids was evaluated by exposing a nanohybrid solution to near-infrared (NIR) light in the presence of DMA (9,10-dimethylanthracene) as a probe for singlet oxygen [21]. DMA (100 μM) in dimethyl sulfoxide (DMSO) was added to ZrMOFs and PD/ZrMOF nanohybrids (1 mg/ml). The samples were exposed directly to a NIR laser (λ = 670 nm, 167 mW/cm^2^, Shanghai Laser & Optics Century Co. Ltd., China) for 0, 1, 2, 3, 4, 5, 10, and 30 min. The fluorescence intensities were measured using a microplate reader (Infinite 200 PRO, Tecan, Wine, Austria) at 420 nm for DMA.

### Photothermal property of nanohybrids

The nanohybrids were tested under 808-nm laser irradiation (750 mW/cm^2^). Samples of ZrMOFs and PD/ZrMOFs (1 mg/ml) were placed in 8-well chamber slides and exposed to an 808-nm laser. Each chamber was observed using a NIR camera (Optris Xi400, Germany), and temperature over time was monitored with the Optris PIX connect software.

### Stability test of nanohybrids

The colloidal stability of ZrMOFs in a physiological buffer after coating with the PD was evaluated by coating the ZrMOFs with the various weight ratios of ZrMOF:DOPA [with a reaction ratio of ZrMOF:DOPA (w/w) of 1:0, 1:1, 1:2, 1:3, 1:4, and 1:5]. The stability test was performed in phosphate-buffered saline (PBS) buffer (pH 7.4) at different time points (0, 0.5, 2, and 72 h). At each time point, the supernatants were collected and visualized after precipitating the nanohybrids.

### pH-sensitive DOX release from nanohybrids

The pH-responsive DOX release from DOX-hyd-PD/ZrMOF nanohybrids was tested by dissolving equal amounts of samples (PD/ZrMOFs, PD/ZrMOFs, and DOX mixture, and DOX-hyd-PD/ZrMOFs, 1 mg/ml) in 3 different media [pH 7.4 PBS buffer, pH 5.0 sodium acetate buffer, and Dulbecco’s modified Eagle’s medium (DMEM) with 10% fetal bovine serum (FBS) and 1% antibiotics] and incubated for 24 h at 37 °C. Subsequently, the nanohybrid samples were centrifugated at 400,000 rpm for 30 min to precipitation. The hydrophilic DOX released from the nanohybrids was collected from the supernatant, and the amount of released DOX was evaluated by measuring the intensity of the fluorescence emission peak of DOX (600 nm) using a microplate reader.

### Cell culture

Mouse colon cancer cells, CT-26, were cultured in DMEM containing 10% FBS and 1% penicillin/streptomycin (antibiotics) and grown in a 5% CO_2_ humidified incubator at 37 °C.

### Cell viability test

A WST (water-soluble tetrazolium) assay was performed to evaluate the cellular toxicity of nanohybrids. Briefly, CT-26 cells (2 × 10^4^/well) were seeded on a 96-well plate and cultured in a 5% CO_2_ incubator at 37 °C overnight. Subsequently, PD/ZrMOF nanohybrids at concentrations of 0, 10, 100, 200, or 500 μg/ml were added to the cell and incubated for 24 h. The cell viabilities were determined by measuring the absorption at 450 nm.

### Cell imaging

The cellular toxicity of nanohybrids was visualized by adding 50 μg/ml of nanohybrids (ZrMOFs or PD/ZrMOFs) to CT-26 cells (5 × 10^4^ cells/well on 8-well chambered coverslips) and incubating them for 4 h. After laser (670 and 808 nm) irradiation of the sample-treated cells, the cells were further stained with propidium iodide (PI) and 4′,6-diamidino-2-phenylindole (DAPI). For detection of ROS generation, ZrMOF- or PD/ZrMOF (10 μg/ml)-treated cells were incubated with 50 μM of singlet oxygen sensor green (SOSG; Thermo Fisher) for 4 h. After laser irradiation into the cell, cells were further stained with Hoechst 33342. Cell imaging was performed under a confocal microscope (FV1000, Olympus, Japan).

### FACS analysis

CT-26 cells (5 × 10^5^) were seeded in RPMI 1640 supplemented with 1% FBS in a 6-well plate and incubated with ZrMOF and PD/ZrMOF (final concentration, 10 μg/ml). After 4 h of incubation, cells were irradiated with laser (670 and 808 nm) and further incubated for 2 h. After fluorescence-activated cell sorting (FITC)–Annexin V/PI staining for 10 min, cells were directly analyzed by FACS machine (CytoFLEX S, Beckman Coulter, USA).

### Colony-forming assay

CT-26 cells were seeded in 12-well plates at a density of 400 cells each well in 1 ml of RPMI 1640 media. After 24 h, the cells were treated with ZrMOF, PD/ZrMOF, and DOX-hyd-PD/ZrMOF samples at doses of 3.125 to 50 μg/ml. After 48 h, the medium was removed and incubated for 5 d. The cells were fixed with 4% paraformaldehyde for 1 h and stained with 0.05% crystal violet for 20 min. The cells were washed twice with PBS, dried in air, and photographed.

### Migration assay

CT-26 cells (2 × 10^4^) were seeded in RPMI 1640 supplemented with 1% FBS in a 6-well plate with the SPL Scar block (SPL Life Sciences, Korea) to produce a defined area. The cells were incubated for 5 h at 37 °C in a humidified atmosphere containing 5% CO_2_, allowing the cells to adhere to the surface. The cells were treated with ZrMOF, PD/ZrMOF, and DOX-hyd-PD/ZrMOF at 2 μg/ml in each block. The SPL Scar block was removed after 12 h, and the cells were replaced with fresh media (1% FBS). The cell migration was observed under an optical microscope at different times from 0 to 48 h. The number of migrated cells was calculated using ImageJ.

### Western blotting

The CT-26 cells were incubated in 6-well plates. After the ZrMOF, PD/ZrMOF, and DOX-hyd-PD/ZrMOF treatment and incubation for 24 h, the cells were washed twice with cold PBS, followed by the addition of lysis buffer (Sigma, X100) supplemented with a proteinase inhibitor cocktail (Roche, 11873580001). The lysates were cleared by centrifugation at 15,000*g* for 10 min at 4 °C, and the protein concentrations were determined using a BCA Protein Assay Kit (Beyotime, P0009). Each sample was mixed with an equal amount of protein and transferred to a polyvinylidene difluoride B (PVDF) membrane. Subsequently, the PVDF membranes were blocked with tris-buffered saline with Tween 20 (TBST) containing 5% skim milk for 1 h and incubated with various primary antibodies against the following: Beclin-1 (Cell Signaling Technology, 3738), Atg7 (Cell Signaling Technology, 2631), LC3B (Cell Signaling Technology, 3868S), p62 (Cell Signaling Technology, 16177), mamalian target of rapamycin (mTOR, Cell signaling Technology, 2972), phospho-mTOR (Cell Signaling Technology, 2971), and β-actin (Cell Signaling Technology, 4970) overnight at 4 °C. Subsequently, the PVDF membranes were washed 3 times with TBST and incubated with horseradish peroxidase (HRP)-labeled secondary antibodies (Cell Signaling Technology) for 1 h, followed by the addition of ECL reagent (Pierce, 32132) to react for 5 min. Immunoblotting was visualized using a LI-COR CLx infrared scanner (Nebraska, USA).

### In vivo pharmacokinetic study

All animal experiments were performed at the Animal Research Laboratory at Inha University and approved by the Inha University Animal Care Committee (IUACC; INHA 201030-732). CT-26 cells (2 × 10^5^) were injected subcutaneously into the back of BALB/c nude mice. After 2 weeks, PBS, ZrMOFs, and PD/ZrMOFs (1 mg/kg) were injected into the mouse tail vein. After 72 h, each mouse images were obtained using an in vivo image analyzer (FOBI, NEO Science, South Korea). The organs (heart, lung, spleen, liver, kidney, and tumor) were harvested, and the fluorescence intensity of each organ was analyzed using ImageJ software.

### Blood circulation of DOX

The pharmacokinetics were evaluated by injecting free DOX (5 mg/kg) and DOX-hyd-PD/ZrMOF nanohybrids (50 mg/kg) intravenously into the tail vain of BALB/c mice (*n* = 3 per each group). After the blood was taken from the eye socket of mice, the collected blood was centrifuged at 1,000 rpm for 5 min to obtain serum, and the fluorescence intensity of DOX (wavelength: ~600 nm) and nanohybrids (wavelength: ~650 nm) was measured using a microplate reader.

### In vivo tumor suppression effect of nanohybrids under laser irradiation

CT-26 cells (2 × 10^5^) were subcutaneously injected into the back of BALB/c mice. When primary tumors reached ~100 mm^3^, the mice were divided into 16 groups (*n* = 3). PBS, ZrMOFs, PD/ZrMOFs, or DOX-hyd-PD/ZrMOF nanohybrids were injected intratumorally at 10 mg/kg. Each tumor region was irradiated with a laser [λ = 671 nm (100 J/cm^2^) and λ = 808 nm (450 J/cm^2^)] once a day for 3 consecutive days. The tumor sizes were measured for 10 d. The mice were sacrificed on day 10 after injecting nanohybrids. The tumors were recovered and analyzed using H&E (hematoxylin and eosin) staining.

### Statistical analysis

Statistical analysis was performed using Origin software. The data were presented as mean ± standard deviation (SD) for all results. The statistical significance was determined using a one-way analysis of variance (ANOVA) with Tukey, with *P < 0.05, **P < 0.01, and ***P < 0.001 considered significant.

## Results

### Preparation and characterization of nanohybrids

**Fig. 2.**
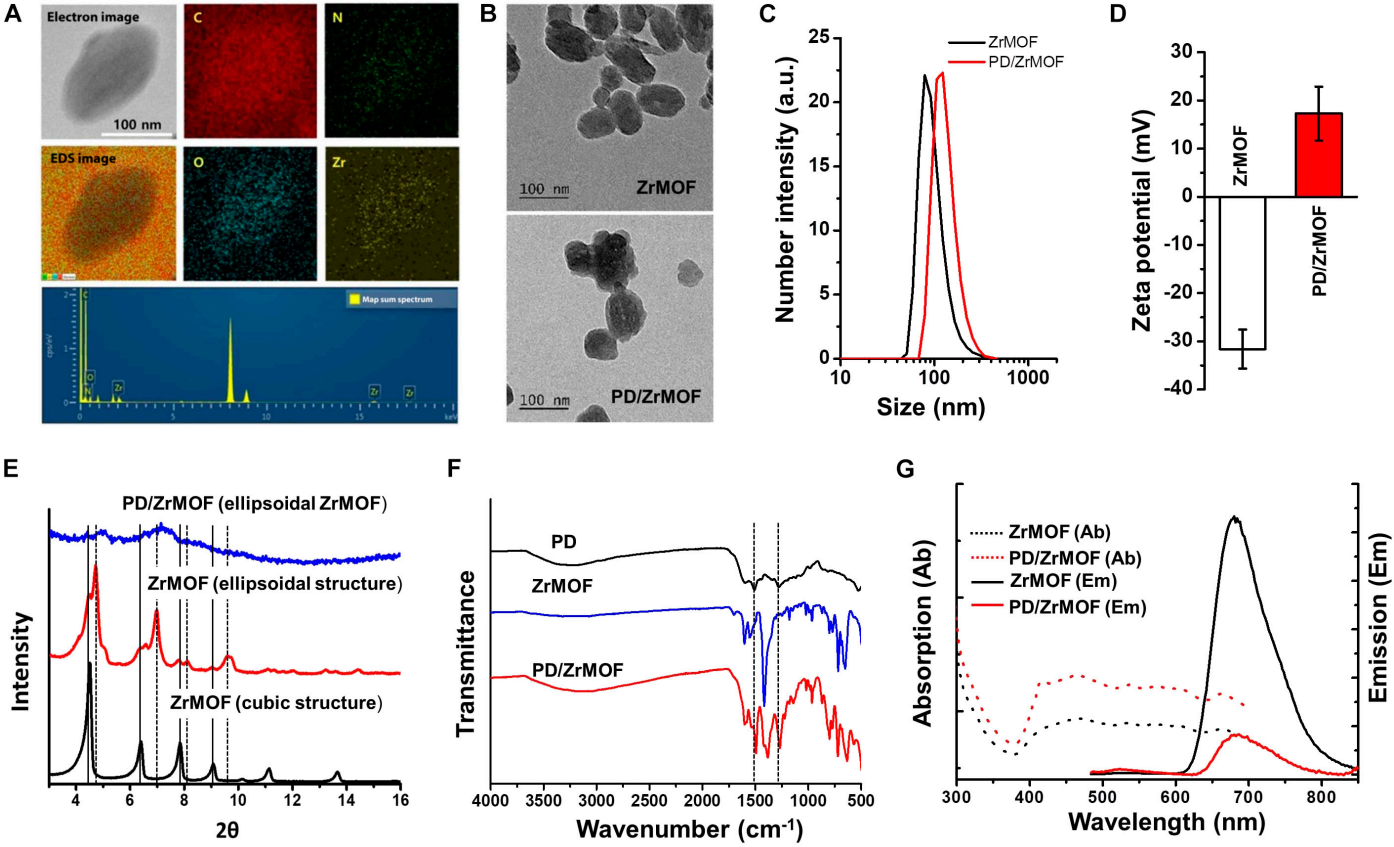
Physicochemical characterization of the nanohybrids. (A) EDS mapping of ZrMOFs. (B) TEM images of ZrMOFs and PD/ZrMOF nanohybrids. Hydrodynamic size (C) and zeta potential (D) of ZrMOFs and PD/ZrMOF nanohybrids determined by DLS analysis (*n* = 3). (E) XRD spectrum of ZrMOFs and PD/ZrMOF nanohybrids. Specific peaks: cubic structure (solid lines) and hexagonal structure (dashed lines). (F) FT-IR spectra of ZrMOFs and PD/ZrMOF nanohybrids. (G) Absorption/emission spectra of ZrMOFs and PD/ZrMOF nanohybrids.

Nano-sized ZrMOFs were produced using the modified synthetic method. The synthesized ZrMOFs were analyzed by energy-dispersive x-ray spectroscopy (EDS). EDS mapping of ZrMOF showed the distribution of carbon (C), nitrogen (N), oxygen (O), and Zr within the nanoparticle (NP). The atomic percentage of each element was measured to 91.3% for C, 0.01% for N, 8.35% for O, and 0.26% for Zr through EDS spectrum analysis (Fig. 2A). The TEM images revealed that the ZrMOF NPs were round or ellipsoidal shape, and the PD-coated ZrMOF (PD/ZrMOF) nanohybrids maintained a similar morphology (Fig. 2B and Fig. S1). Hydrodynamic particle size and zeta potential value of nanohybrids were measured by DLS. Hydrodynamic size and zeta potential of ZrMOFs were 104.8 ± 38.7 nm and −31.6 ± 4.1 mV with a narrow distribution (PDI = 0.202), respectively. After PD coating, the hydrodynamic size was increased by 132.2 ± 40.6 nm with a narrow distribution (PDI = 0.165) (Fig. 2C). In addition, the zeta potential was converted to a positively charged form, 17.3 ± 5.6 mV (Fig. 2D). As shown in Fig. 2E, the x-ray crystallography (XRD) peaks of our ZrMOF with an ellipsoidal structure indicate that the material has 2 phases: Zr-based porphyrinic MOF-525 (cubic structure, 2θ = 4.5, 6.3, 7.9, 9.0) and PCN-223 (hexagonal structure, 2θ = 4.8, 7.0, 8.1, 9.7), consistent with a previous report [22].

As shown in Fig. 2F, Fourier transform infrared (FT-IR) analysis revealed that specific peaks (C=N, 1,510 cm^−1^ and C–N–C, 1,333 cm^−1^) of PD appeared in PD/ZrMOF nanohybrids. Especially, as the PD contents increased, these peaks showed more strong peaks due to the high PD content (data not shown). We also measured the absorption/emission spectrum of ZrMOF and PD/ZrMOF nanohybrids (Fig. 2G). The results indicate that porphyrin was successfully incorporated into the synthesized ZrMOFs, showing a strong fluorescent peak at ~680 nm. However, PD-coated ZrMOF nanohybrids exhibited fluorescence quenching due to the broad absorption of PD.

The pH-sensitive drug release property was introduced by conjugating DOX with 3,3′-dithiobis (propanoic dihydrazide). Thiolated DOX-hyd was produced by DTT (Fig. S2). Synthesized DOX-hyd was analyzed by ^1^H nuclear magnetic resonance (NMR) spectroscopy, which showed specific peaks (−CH_2_− of hydrazine linker) at 2.48 and 2.84 parts per million (ppm) (Fig. S3).

**Fig. 3.**
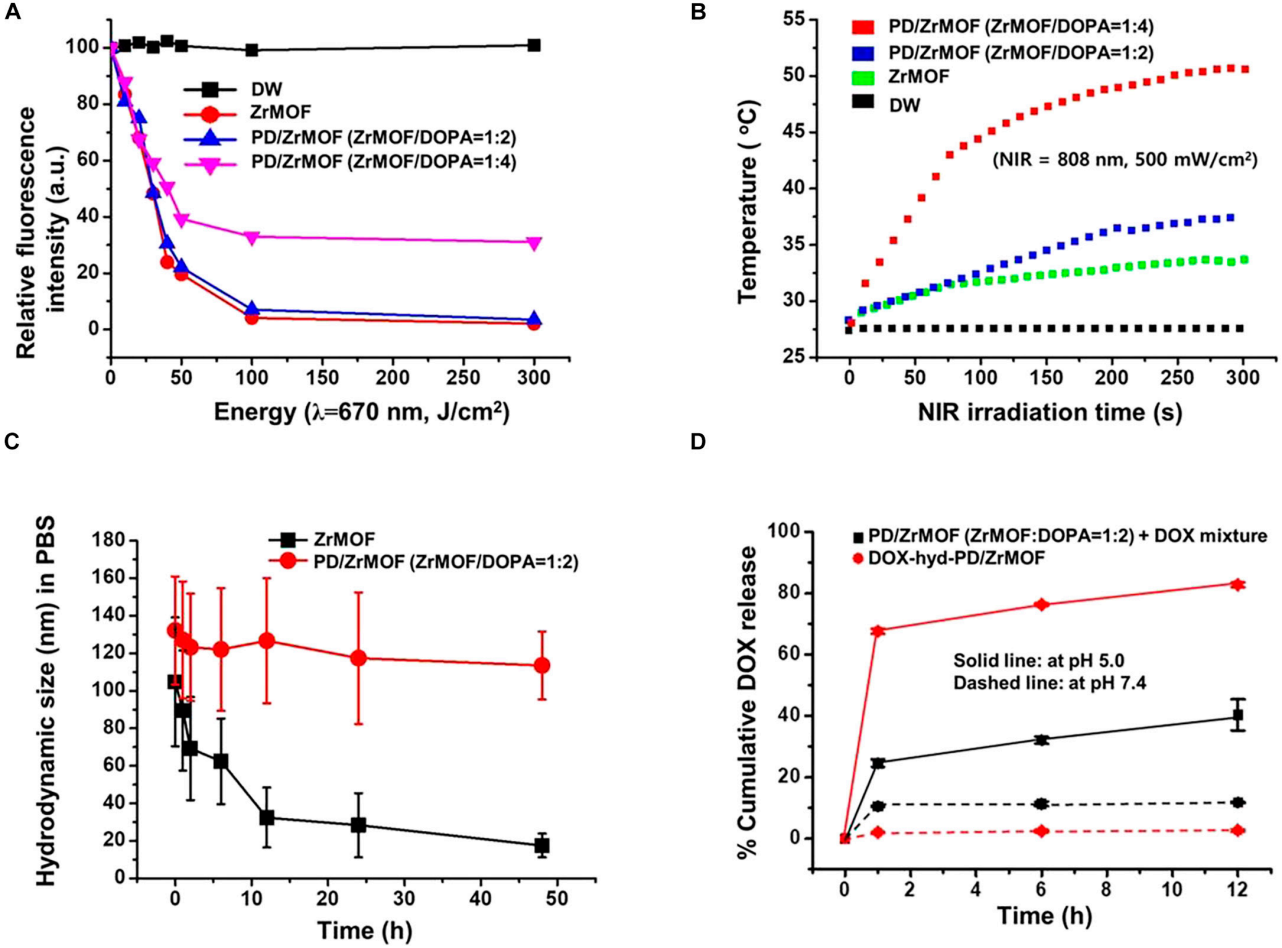
Photodynamic and photothermal characterization of the nanohybrids. (A) Singlet oxygen generation test using DMA under 670-nm laser irradiation. (B) Time-dependent temperature changes; 808-nm laser irradiation (750 mW/cm^2^). (C) Time-dependent stability of ZrMOF and PD/ZrMOF (ZrMOF/DOPA = 1:2) nanohybrids in PBS buffer (pH 7.4) measured using DLS (*n* = 3). (D) Cumulative DOX release from nanohybrids at PBS buffer (pH 7.4) and sodium acetate buffer (pH 5.0) (*n* = 3).

We confirmed the singlet oxygen generation of nanohybrids upon irradiation of NIR laser (670 nm) in the presence of a fluorescent singlet oxygen probe, 9,10-dimetylantracene (DMA) (Fig. 3A). The fluorescence intensity of DMA decreases in an irradiation energy-dependent manner due to conversion of ROS-derived endoperoxide anthracene. For 3 samples, ZrMOF and 2 PD/ZrMOFs hybrids with ZrMOF:DOPA = 1:2 and 1:4 reaction ratios, the fluorescence of DMA (at 430 nm) decreased as the NIR exposure increased. The fluorescence quenching effects of DMA were similar for both ZrMOF and PD/ZrMOF (ZrMOF:DOPA = 1:2), achieving over 95% quenching at 100 J/cm^2^. However, in the case of PD/ZrMOF (ZrMOF:DOPA = 1:4), the fluorescence quenching effect of DMA was relatively lower, at approximately 70%. This is attributed to the thicker PD layer coated on the ZrMOF NPs, which is expected to reduce ROS generation by the TCPP inside. Based on these results, we proceeded with in vitro and in vivo studies under the ZrMOF:DOPA = 1:2 ratio, which minimized the suppression of the PDT effect caused by the PD coating.

The PD-coated nanohybrids, PD/ZrMOF, can show photothermal properties (Fig. 3B). Nanohybrid exposure to an 808-nm laser (500 mW) and the temperature were monitored. The temperature of distilled water without nanohybrids and ZrMOF solution was slightly increased by ~7 °C at 300 s after light irradiation. On the other hand, the temperature of the PD/ZrMOF (ZrMOF:DOPA = 1:2) nanohybrids increased to >10 °C, while the temperature of the PD/ZrMOF (ZrMOF:DOPA = 1:4) nanohybrids increased to >23 °C. This finding indicates that the photothermal effect becomes more pronounced as the PD content increases.

ZrMOFs can be easily hydrolyzed and degraded in the aqueous solution due to the weak bonding energy between metal ions and organic compounds [23–25]. The PD was introduced as a surface coating material to improve the problem, and the colloidal stability under physiological buffer conditions was tested. As shown in Fig. S4, ZrMOFs were reacted with different amounts of dopamine (DOPA, PD) (ZrMOF:DOPA = 1:0, 1:1, 1:2, 1:3, 1:4, and 1:5, w/w). Each sample was added to the PBS buffer solution, and the time-dependent degradation of nanohybrids was tested. In the case of ZrMOFs and PD/ZrMOF (ZrMOF:DOPA = 1:1), the nanohybrids were easily degraded and solubilized in the supernatant at 0.5 h. On the other hand, the PD/ZrMOF (ZrMOF:DOPA in ratios ranging from 1:2 to 1:5) nanohybrids showed hydrolysis resistance and remarkably improved colloidal stability in the PBS buffer for 72 h. The stability of the PD/ZrMOF nanohybrids in PBS was also confirmed by DLS analysis. As shown in Fig. 3C, the size of the ZrMOF continuously decreased over time, while the PD/ZrMOF (ZrMOF:DOPA = 1:2) nanohybrids exhibited almost the same size over 48 h.

DOX with pH-cleavable thiolated hydrzone moiety was further conjugated with the PD/ZrMOF nanohybrids. Our results revealed that the DOX-hyd (Fig. S2) and PD/ZrMOF conjugation accounted for 13.35 wt % based on absorption spectroscopic analysis (data not shown).

To evaluate the pH-dependent DOX release, PD/ZrMOF plus DOX mixture and DOX-hyd-PD/ZrMOF were added to different buffer solutions (pH 7.4 PBS and pH 5.0 sodium acetate buffer), and time-dependent accumulated DOX release was analyzed through the absorption spectrum of free DOX in solution after removal of nanohybrids by centrifugation. As shown in Fig. 3D, no noticeable DOX release occurred from the PD/ZrMOF plus DOX mixture or DOX-hyd-PD/ZrMOF nanohybrids in the PBS (pH 7.4). On the other hand, the DOX-hyd-PD/ZrMOF nanohybrids showed significantly higher DOX release in the acidic sodium acetate buffer (pH 5.0) compared to the mixture sample.

### Cellular toxicity of nanohybrids

**Fig. 4.**
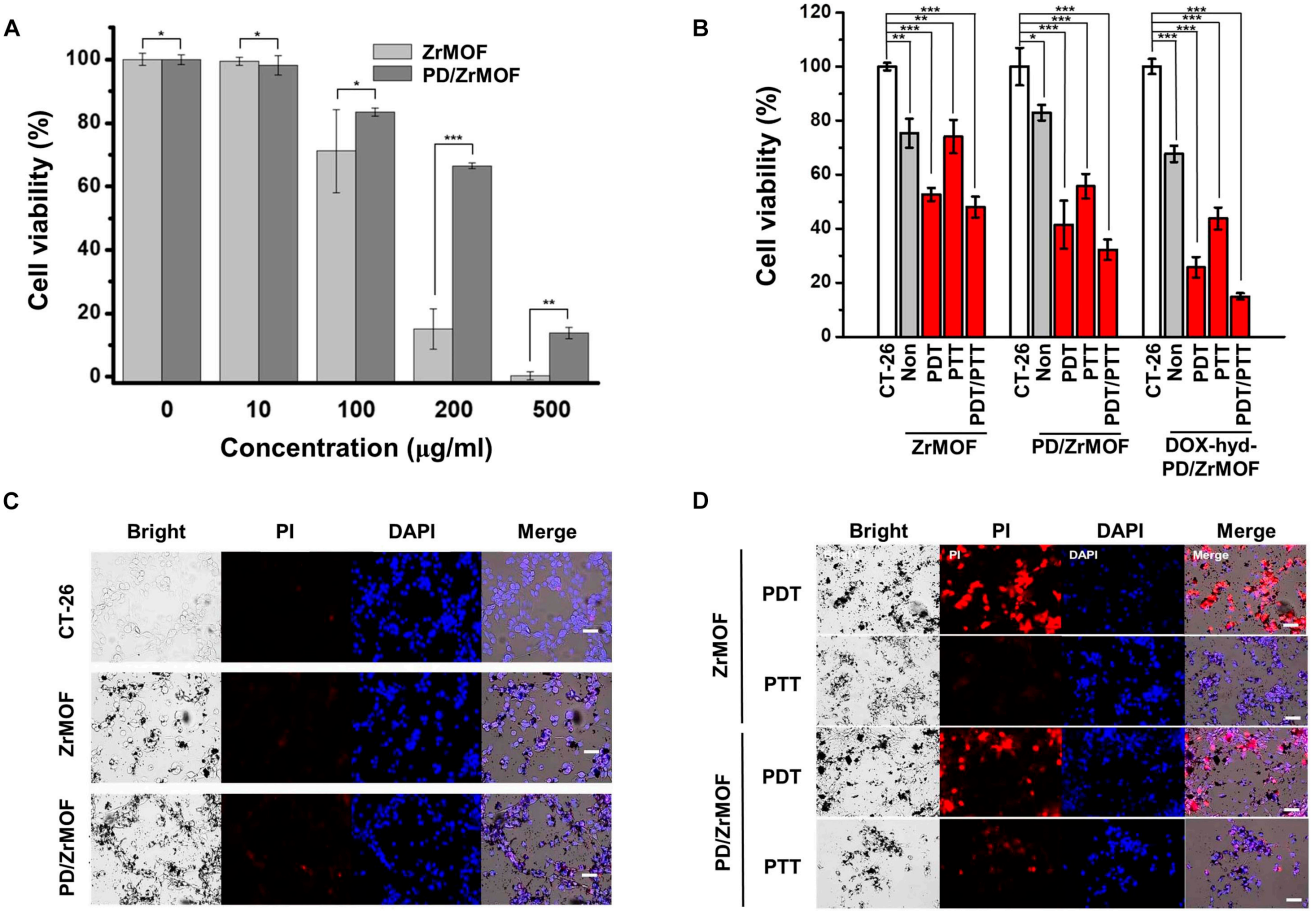
Cell viability of CT-26 cells incubated with various concentration of nanohybrids (A) without and (B) with laser (PDT, PTT, and PDT/PTT) irradiation (***P < 0.001, **P < 0.005, *P < 0.01, *n* = 3). Confocal microscopic images of CT-26 cells cultured with ZrMOF and PD/ZrMOF without (C) and with (D) laser irradiation. The cells were stained with PI and DAPI for dead cells and nuclei, respectively. Scale bar, 100 μm.

The cellular compatibility of ZrMOFs and PD/ZrMOF nanohybrids was evaluated against CT-26 cells. The ZrMOF and PD/ZrMOF nanohybrids at 10 μg/ml did not affect the cell viability, as shown in Fig. 4A. At concentrations of 100 μg/ml and higher, however, the cellular toxicity of PD/ZrMOF nanohybrids was significantly lower than that of ZrMOFs. Even at a high concentration of 200 μg/ml, the cell viability of the PD/ZrMOF nanohybrids remained around 70%, suggesting that the coating of PD improves the cytocompatibility of ZrMOFs. In addition, the photo-induced cellular toxicity test was performed in the presence of a 670-nm laser for PDT and an 808-nm laser for PTT. The CT-26 cells were incubated with the nanohybrids (ZrMOF, PD/ZrMOF, and DOX-hyd-PD/ZrMOF, final concentration of each nanohybrids = 50 μM). After light irradiation, the cells were further incubated for 24 h, and the cell viability was also tested. The cells treated with ZrMOF showed higher toxicity only during PDT and a combination of PDT and PTT, as shown in Fig. 4B. In the case of PD/ZrMOF and DOX-hyd-PD/ZrMOF nanohybrid treatment, all cells showed enhanced toxicity in the presence of PDT, PTT, and PDT/PTT combination compared to the ZrMOF groups. In particular, the cells treated with DOX-hyd-PD/ZrMOF showed the greatest cytotoxicity because of the effects of DOX release compared with that of PD/ZrMOF.

The photo-induced cellular toxicity of the nanohybrids was visualized by confocal microscopy. In the absence of irradiation, CT-26 cells treated with ZrMOFs or PD/ZrMOF nanohybrids showed no fluorescence in the PI channel (Fig. 4C). On the other hand, after irradiation with a 670- or 808-nm laser, only the 670-nm laser-irradiated ZrMOFs and PD/ZrMOF nanohybrids showed remarkable red fluorescence, indicating cell death (Fig. 4D). The PTT effect of PD/ZrMOF resulted in slight red fluorescence upon 808-nm laser irradiation compared to no fluorescence in ZrMOF.

**Fig. 5.**
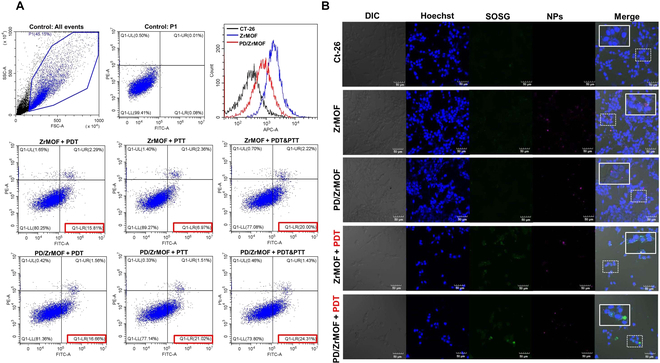
Flow cytometry and cellular imaging. (A) Cellular uptake of ZrMOF and PD/ZrMOF nanohybrids, and apoptosis by laser irradiation; PDT (670 nm), PTT (808 nm), and PDT/PTT (670 nm + 808 nm). Apoptosis measurement from FITC-Annexin V (FITC-A) versus PI (PE-A) florescence distribution of CT-26 cells (10,000) within the P1 region. Red box in the images indicates early apoptosis. (B) Confocal microscopic images of CT-26 cells treated with ZrMOF and PD/ZrMOF nanohybrids; SOSG (ROS probe) fluorescence changes were observed with/without PDT (670 nm) irradiation. White square box, magnification of the dashed square area. Scale bar, 50 μm.

To evaluate the cellular uptake and apoptosis of nanohybrids, flow cytometry analysis was performed. CT-26 cells were incubated with 10 μg/ml of ZrMOF and PD/ZrMOF nanohybrids for 4 h, followed by PDT (670 nm), PTT (808 nm), and PDT/PTT (670/808 nm) irradiation. Then, FITC-Annexin V/PI were further stained. As shown in Fig. 5A, the fluorescence intensity at allophycocyanin (APC) channel (660 ± 20 nm) increased due to the cellular uptake of NPs. However, the fluorescence intensity of ZrMOF was stronger than that of PD/ZrMOF nanohybrids. This suggests that the fluorescence quenching effect is due to the PD coating in the PD/ZrMOF nanohybrids, rather than to reduced cellular uptake. Apoptosis analysis using FITC-Annexin V/PI staining showed early apoptosis effects of 15.81% for PDT, 6.97% for PTT, and 20% for PDT/PTT in the ZrMOF-treated samples. For PD/ZrMOF, the early apoptosis effects were 16.66%, 21.02%, and 24.31%, respectively. These results indicate that ZrMOF primarily influences cell death through PDT rather than PTT, while PD/ZrMOF appears to be affected by both PDT and PTT.

Furthermore, to examine the cellular ROS generation induced by PDT, fluorescence images were obtained using confocal microscopy with SOSG staining. As shown in Fig. 5B, samples treated with only ZrMOF or PD/ZrMOF without PDT displayed only the red fluorescence in the NP channel, with no green SOSG fluorescence observed. In contrast, when PDT was applied, green fluorescence was detached in the cytosol region of CT-26 cells in both ZrMOF and PD/ZrMOF treatments.

**Fig. 6.**
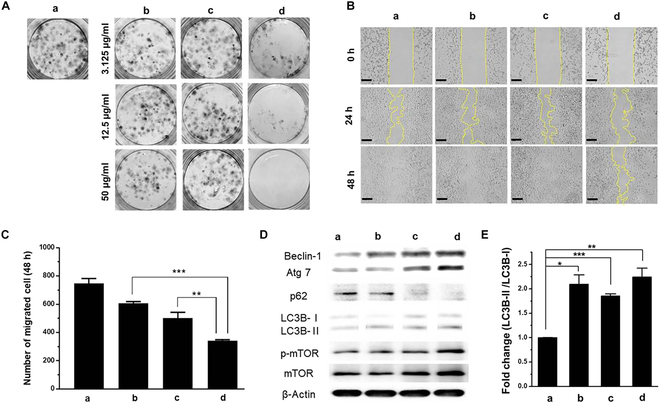
Proliferation and migration study of CT-26 colon cancer (A to C) and autophagy induction after exposure to ZrMOFs (D and E): (a) nontreated, (b) ZrMOF, (c) PD/ZrMOF, and (d) DOX-hyd-PD/ZrMOF. (A) Colony-forming assay of CT-26 cells treated with the NPs with different concentrations for 48 h. (B) Time-dependent cell migration images (NP concentration = 2 μg/ml). Scale bar, 200 μm. (C) Migrated cell numbers at 48 h measured by ImageJ (***P < 0.001, **P < 0.002, *n* = 3). (D) Western blot of autophagy-related protein of Beclin-1, Atg7, p62, LC3, p-mTOR, and mTOR. (E) Fold change of LC3B-ll /LC3B-l analyzed from Western blot. (***P < 0.001, **P < 0.005, *P < 0.01, *n* = 3).

Cell colony formation and cell migration are important processes in colon cancer tumorigenesis and metastasis. An in vitro assay was performed to evaluate the effects of nanohybrids on the proliferation and migration of CT-26 cells. Colony formation of CT-26 cells treated with MOF and PD/MOF (3.125, 12.5, and 50 μg/ml) showed slightly lower clustering than the cell control, as shown in Fig. 6A. On the other hand, in the case of DOX-hyd-PD/ZrMOF treatment, CT-26 colony formation showed a prominent decrease in a dose-dependent manner. In addition, the would-healing assay (Fig. 6B) revealed a decrease in the migration ability of DOX-hyd-PD/ZrMOF-treated colon cancer cells, and the estimated number of cells infiltrated in each ROI area was ~720 (control), ~600 (MOF), ~500 (PD/MOF), and ~350 (DOX-hyd-PD/ZrMOF) (Fig. 6C). These results confirmed that this nanohybrid exhibits excellent inhibitory effects on cell growth and movement.

### Autophagy regulation by nanohybrids

Immunoblotting was performed on cells treated with ZrMOF, PD/ZrMOF, and DOX-hyd-PD/ZrMOF nanohybrids to assess the regulation of autophagy in CT-26 cells (Fig. 6D and E). Beclin-1, a key factor in early-stage autophagy initiation and phagophore assembly, was up-regulated in all treatments compared to the control [24]. This phenomenon was confirmed by the elevation of the autophagy-related gene Atg7, which acts as a ubiquitin-E1-like enzyme mediating autophagosomal processing [26]. Furthermore, the conversion of endogenous LC3B-I to the lipidated form LC3B-II (LC3B-phosphatidylethanolamine) was accelerated in the ZrMOF, PD/ZrMOF, and DOX-hyd-PD/ZrMOF treatments, revealing an increase in the number of autophagosomes in the cancer cells (Fig. 6E). The abundance of sequestosome 1 (p62), a specific polyubiquitin-binding protein recruited to autophagosomes and degraded in autolysosomes, was similar in the MOF group and control [27]. In contrast, p62 expression in the nanohybrid groups (PD/ZrMOF and DOX-hyd-PD/ZrMOF) was noticeably down-regulated, indicating ongoing autophagy. However, ZrMOF treatment did not affect mTOR signaling. As shown in Fig. S5, an analysis of activated mTOR (p-mTOR), a key upstream regulator involved in autophagy inhibition, revealed that the p-mTOR/mTOR ratio increased compared to the control. In contrast, PD/ZrMOF treatment reduced the p-mTOR/mTOR levels relative to the control, indicating its impact on autophagy activation. DOX-hyd-PD/ZrMOF treatment increased the p-mTOR/mTOR levels again compared to the control, demonstrating effects like those of ZrMOF treatment. These findings contrast with the previously known mTOR signaling–autophagy axis.

### In vivo study

**Fig. 7.**
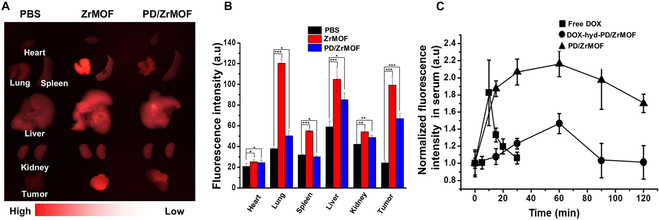
Pharmacokinetic study. (A) Fluorescence images of various organs (heart, lung, spleen, liver, kidney, and tumor) harvested at 72 h after the intravenous injection of nanohybrids into subcutaneous CT-26 tumor-bearing mouse. (B) Fluorescence intensity of tissues analyzed by ImageJ (***P < 0.001, **P < 0.005, *P < 0.01, *n* = 3). (C) Blood circulation of profiles of free DOX and DOX-hyd-PD/ZrMOF nanohybrids (■, free DOX; ●, DOX-hyd-PD/DOX; the normalized fluorescence intensity of DOX in mouse serum was measured at λ = 600 nm) (*n* = 3). Florescence of PD/ZrMOF (▲) was measured at λ = 650 nm (*n* = 3).

The in vivo biodistribution of nanohybrids was evaluated in CT-26 cancer-bearing BALB/c nude mice. Two weeks after a subcutaneous injection of CT-26 into mice, ZrMOFs and PD/ZrMOF nanohybrids were injected into the tail vein. After 72-h maintenance, each organ (heart, lung, spleen, liver, kidney, and tumor) was extracted from the mice to analyze more detailed in vivo nanohybrid distribution by fluorescence imaging (Fig. 7A). Furthermore, the relative fluorescence intensity was measured by image analysis (Fig. 7B). The fluorescence intensity graph of various organs showed that the fluorescence intensity of tumor region in the ZrMOF-treated groups was ~4 times greater than that of PBS-treated groups. Although the fluorescence intensity in the PD/ZrMOF nanohybrid-treated group was slightly lower than that of the ZrMOF-treated group because of fluorescence quenching by PD, the fluorescence intensity of the PD/ZrMOF nanohybrid-treated group also showed ~2.7 times greater than that of control PBS-treated group.

The pharmacokinetic properties of the nanohybrids were examined by injecting the DOX-hyd-PD/ZrMOF nanohybrids and free DOX intravenously into the mouse tail vein and collecting blood at different time points to monitor changes in the concentration of nanohybrids and free DOX. The free DOX showed a very short blood circulation time (maximum peak at ~10 min), as shown in Fig. 7C. In contrast, the DOX-hyd-PD/ZrMOF nanohybrids showed sustained blood circulation (maximum peak at ~60 min) compared to the free DOX treatment.

The in vivo therapeutic efficacy of DOX-hyd-PD/ZrMOF nanohybrids for PDT/PTT and chemotherapy was investigated using the CT-26 tumor-bearing mouse model. After tumor growth to ~100 mm^3^, the same amounts of ZrMOFs, PD/ZrMOF, or DOX-hyd-PD/ZrMOF nanohybrids (dosage 10 mg/kg) were intratumorally injected. Subsequently, the tumors were irradiated with a 670-nm laser (100 J/cm^2^, PDT), 808-nm laser (450 J/cm^2^, PTT), and dual laser (670 nm + 808 nm, PDT/PTT) once daily for 3 consecutive days.

**Fig. 8.**
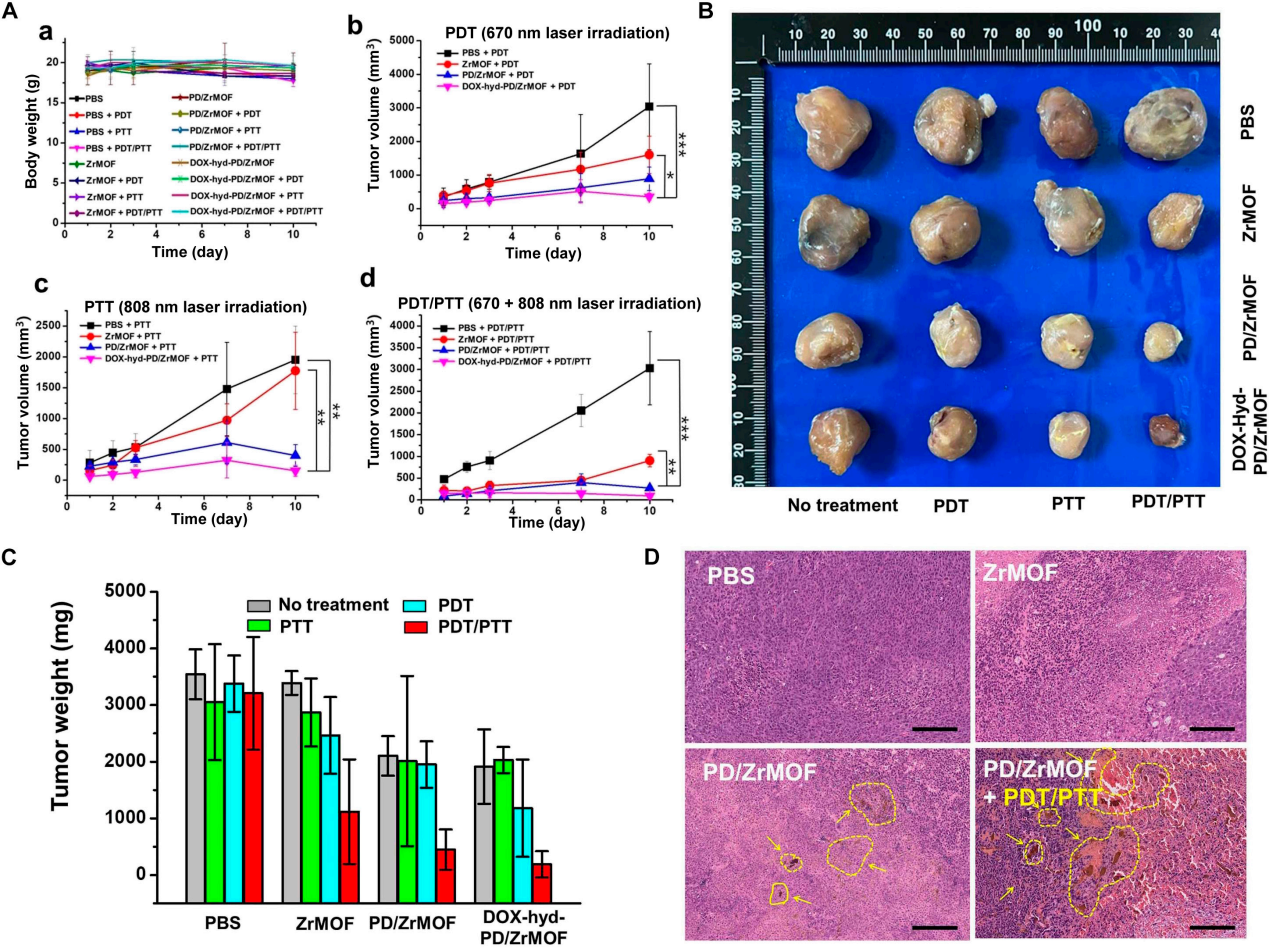
In vivo therapeutic effect of nanohybrids. (A) Body weight and tumor volume change measurements (*P < 0.05, **P < 0.02, ***P < 0.001). (a) Body weights of BALB/c mice. Tumor volume changes over time under PDT (b), PTT (c), and PDT/PTT (d) condition (*n* = 3). (B) Image of tumor tissues excised after 10 d. (C) Tumor weights of excised tumor tissues (*n* = 4). (D) H&E staining images of tumor tissues. Yellow arrows indicate PD/ZrMOF-infiltrated tumor regions. Scale bar, 200 μm.

Under all experimental conditions, the body weights of the mice remained unchanged (Fig. 8A, a), and the tumor volume in the control PBS group gradually increased over 10 d, regardless of PDT or PTT treatment (Fig. 8A, b to d). In contrast, the PD/ZrMOF and DOX-hyd-PD/ZrMOF nanohybrid-treated groups showed significantly suppressed tumor growth after laser irradiation (PDT, PTT, and PDT/PTT). Especially, the DOX-hyd-PD/ZrMOF nanohybrids exhibited the best suppression effects under PDT + PTT dual treatment condition with approximately 33 times smaller than the PBS group (Fig. 8A, d) after 10 d. After excising the tumors, each tumor image was obtained, and it was clearly confirmed that the tumor treated with PDT/PTT in DOX-hyd-PD/ZrMOF was significantly smaller compared to the other samples (Fig. 8B). This result was further confirmed by measuring the tumor weights in the graph (Fig. 8C). Histological analysis of the tumors, stained with H&E, revealed that the PDT and PTT combination group (PD/ZrMOF nanohybrids) exhibited a higher level of apoptosis and necrosis compared to the PBS, ZrMOF, and PD/ZrMOF nanohybrids without laser irradiation (Fig. 8D). In the PD/ZrMOF nanohybrid-infiltrated tumor regions (yellow arrows), the cells in the tumor tissue were evenly distributed in the absence of laser irradiation. However, after PDT and PTT treatment, the cells surrounding the NPs were severely destroyed, resulting in tissue damage. In the case of ZrMOF treatment, NPs were not observed in the tumor tissue due to their instability and degradation.

## Discussion

In this study, our synthesized ZrMOFs showed much smaller particle size than those of the previously reported Zr-based porphyrinic MOFs (Fig. S6) [28]. Moreover, these ellipsoidal-shaped ZrMOFs exhibit less aggregation compared to cubic-structured NPs, making them more efficient for biological applications. To load a drug with controlled release properties onto NPs, we synthesized DOX-hyd and incorporated it into the PD/ZrMOF nanohybrids. The results of pH-sensitive DOX release (Fig. 3D) indicates that the PD/ZrMOF nanohybrids can successfully capture DOX-hyd through Schiff base formation and Michael-type addition reactions between the PD and the primary thiol group of DOX-hyd [19]. In the acidic microenvironment, the release of DOX is facilitated by the increased solubility induced by the inherent chemical property of DOX and cleavage of hydrazone of DOX-hyd.

Moreover, we clearly showed the photo-induced cellular toxicity of the nanohybrids from Fig. 4D. The PD/ZrMOF nanohybrids exhibited dual cytotoxicity under irradiation with a 670- and 808-nm laser, whereas the ZrMOF showed cytotoxicity only under 670-nm laser irradiation. These findings indicate that the newly developed PD/ZrMOF nanohybrids can be used as a powerful photodynamic and photothermal agent, and the PD coating mainly contributes to the photothermal effect.

In the result of Western blotting data in Fig. 6D, the significant increase in Beclin-1 levels with the DOX-hyd-PD/ZrMOF treatment compared to the control suggests potent autophagy induction despite the partial inhibition of mTOR activity. The mammalian target of rapamycin (mTOR) is known to promote most anabolic processes within the cell, including the synthesis of proteins, lipids, cholesterol, and nucleotides, while simultaneously increasing extracellular nutrient uptake and suppressing autophagic catabolism [29,30]. However, our findings collectively suggest that the observed autophagy induction may involve mTOR-independent pathways, particularly with a DOX-hyd-PD/ZrMOF treatment, potentially contributing to its efficacy in promoting autophagy in CT-26 cells. This conclusion is supported by the roles of Beclin-1 and ATG7 in the pathways that do not require mTOR inhibition, suggesting that the observed autophagy induction may involve alternative mechanisms independent of mTOR signaling [31,32]. The up-regulation of Beclin-1 and ATG7 suggested the robust activation of autophagy pathways that can operate even in the presence of active mTOR signaling, supporting the notion of mTOR-independent autophagy induction in the experimental context.

In vivo study demonstrated the cancer-specific targeting properties and excellent therapeutic efficacy of the nanohybrids. As shown in Figs. 6B and 7A, ZrMOF and DOX-hyd-PD/ZrMOF nanohybrids were confirmed to accumulate more in tumor tissues than in other organs after 72 h, likely due to the enhanced permeability and retention (EPR) effect [33], which is attributed to the ~100-nm size of the nanohybrids. Furthermore, in Fig. 8A, d, the triple-mode combination group of the DOX-hyd-PD/ZrMOF nanohybrids showed the best cancer therapeutic performance. Thus, combining porphyrinic ZrMOF nanomaterials and smart cancer medicine delivery in a single option provides a powerful and versatile anticancer treatment.

## Conclusion

Multimodal DOX-hyd-PD/ZrMOF nanohybrids through the assembly of Zr/porphyrin clusters, coating of PD, and covalent attachment onto the PD layer were developed for efficient cancer treatment. In particular, the PD coating of the ZrMOFs provided enhanced biocompatibility and colloidal stability. The nanohybrids enabled the simultaneous generation of ROS, such as singlet oxygen, for photodynamic activity and showed photothermal capability for PTT upon NIR laser irradiation. In addition, the nanohybrids displayed pH-responsive anticancer drug delivery capability through the cleavage of the hydrazone between DOX and nanohybrids. In addition, ZrMOFs, PD/ZrMOF, and DOX-hyd-PD/ZrMOF could activate autophagy, suggesting that these materials can be used to regulate autophagy and the effectiveness and evaluation of MOFs. Moreover, the DOX-hyd-PD/ZrMOF nanohybrids under NIR laser irradiation efficiently suppressed in vitro and in vivo cancer growth with tri-mode combinative therapeutic capabilities, including PDT, PTT, and chemotherapy. This study contributes to the design and development of a versatile “three-in-one” nanoplatform that possesses cancer imaging and multiple-mode combination treatment capabilities.

## Data Availability

The authors do not have permission to share data.

## Supplementary Materials

Supplementary 1 Graphical Abstract Figs. S1 to S6
