# Supplementary material for: Autophagy-Regulating, Photothermal Polydopamine-Coated, and Photodynamic Zirconium/Porphyrin-Framed Metal–Organic Frameworks for Enhanced Doxorubicin Therapy in Colon Cancer
Source: Biomater Res. 2025 Jun 12;29:0218. doi: 10.34133/bmr.0218 (PMC12160315; doi:10.34133/bmr.0218)
Supplement: Supplementary 1 — Graphical Abstract Figs. S1 to S6 [file bmr.0218.f1.zip › 3. MOF Supporting Information for revision-2025.04.16.docx]

Supporting Information

**Autophagy-Regulating, Photothermal Polydopamine-Coated, and Photodynamic Zr/Porphyrin-Framed Metal-Organic Frameworks (MOFs) for Enhanced Doxorubicin Therapy in Colon Cancer**

Junghan Lee ^a,1^, Kwangsun Yu ^b,d,1^, Enkhzaya Davaa ^a^, Ratchapol Jenjob ^a^, Phuong Hoa Tran ^a^, Dahee Ryu ^a^, Jongyoon Kim ^a^, Seongju Lee ^a^, Zheyu Shen ^c^, Wha-Seung Ahn ^d^,

Chung-Sung Lee ^e^ and Su-Geun Yang ^a,*^


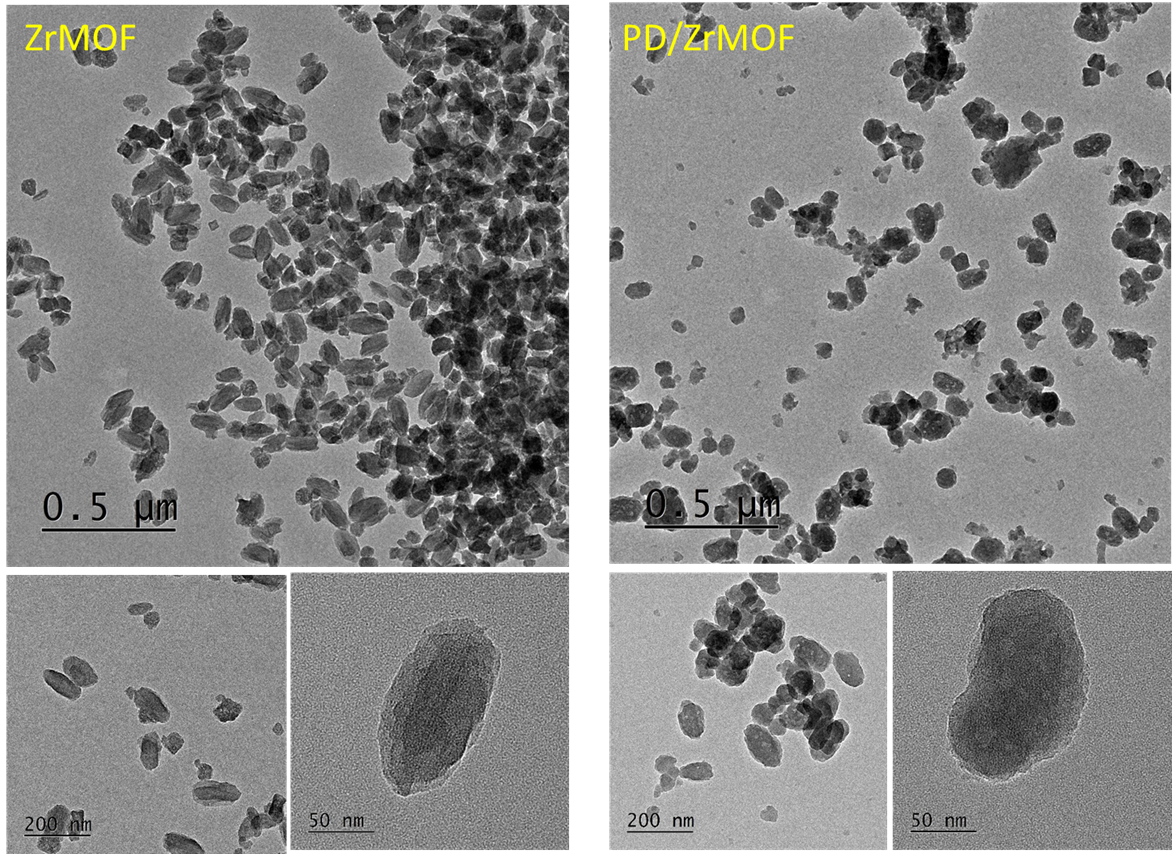


**Fig. S1** TEM images of ZrMOFs and PD/ZrMOF nanohybrids.


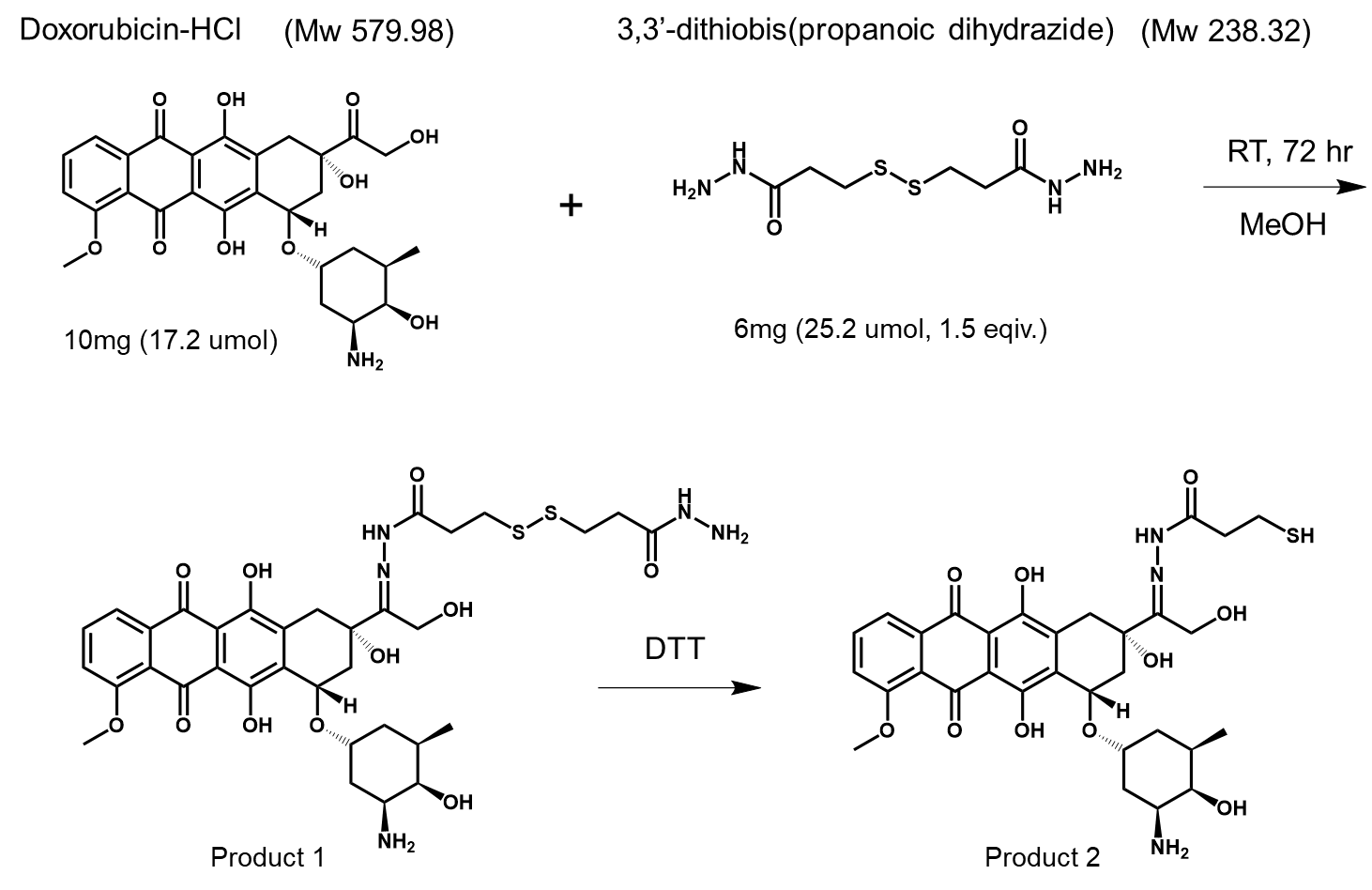


**Fig. S2** Synthetic routes of hydrazine-modified doxorubicin (DOX-hyd).


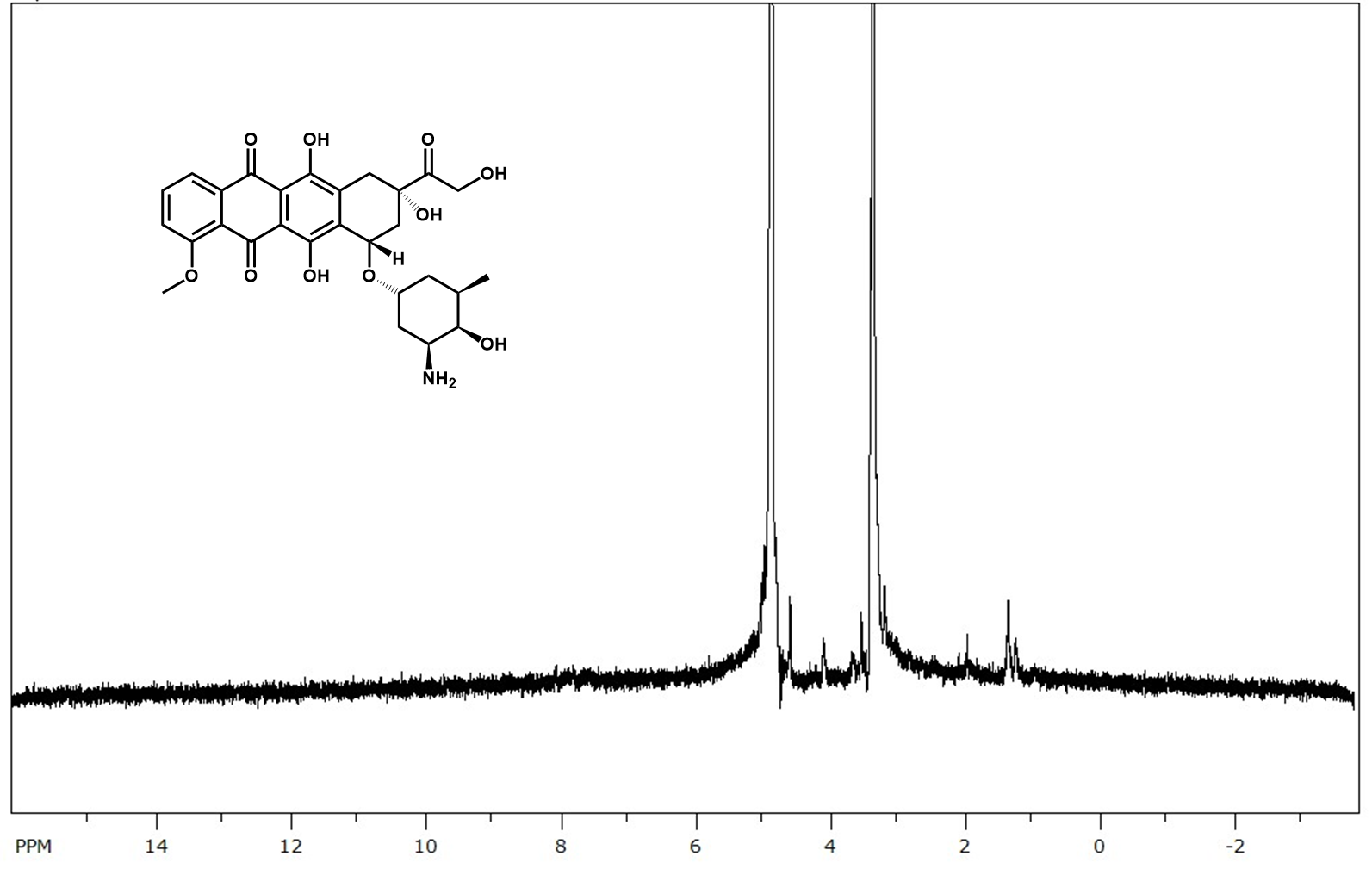


Chemical shift


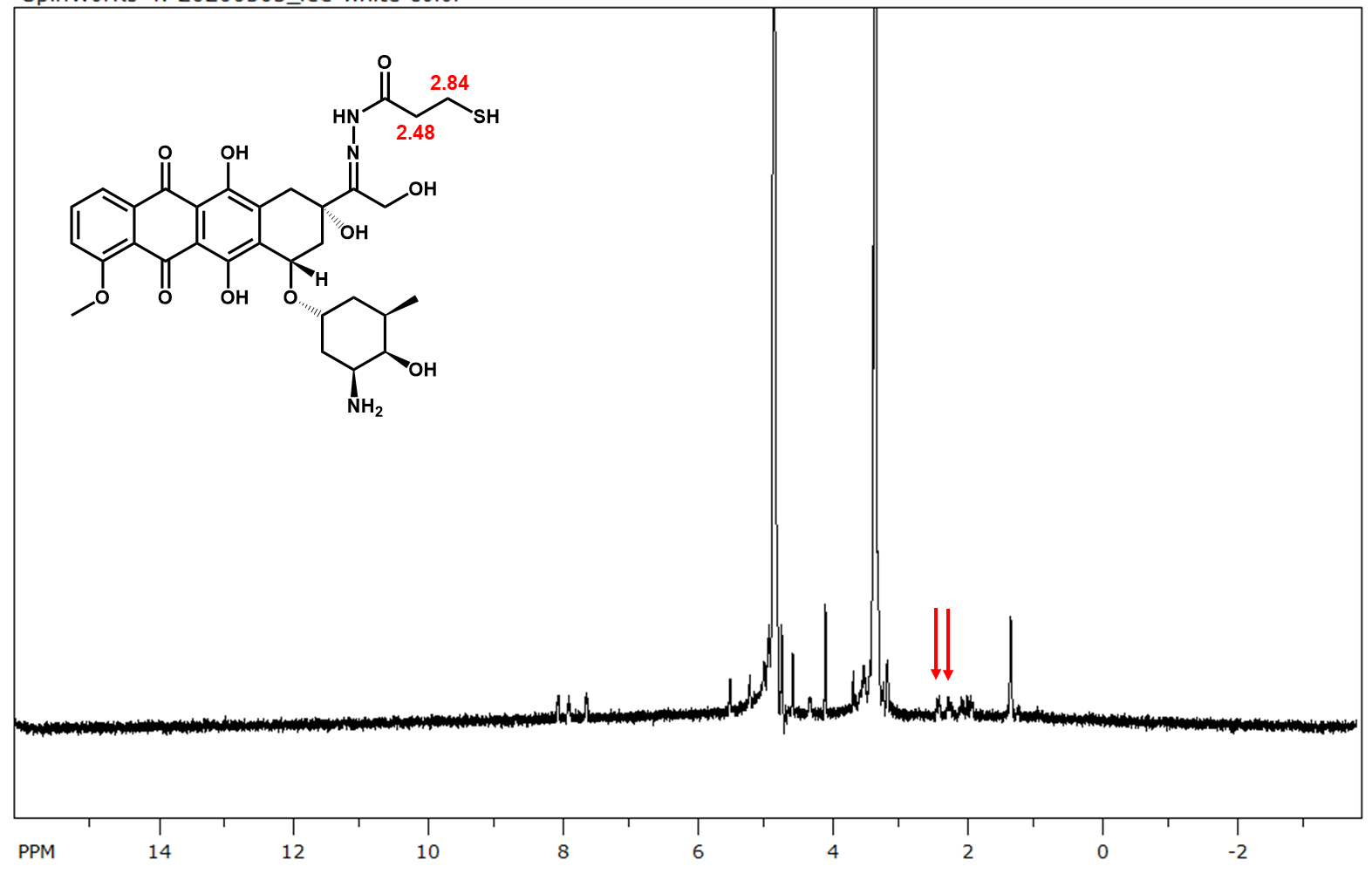


Chemical shift

**Fig. S3** ^1^H-NMR spectra for doxorubicin (DOX) and DOX-hyd in D_2_O; DOX-hyd δ 2.48 (-CH_2_-, 2H), δ 2.84 (-CH_2_-, 2H).


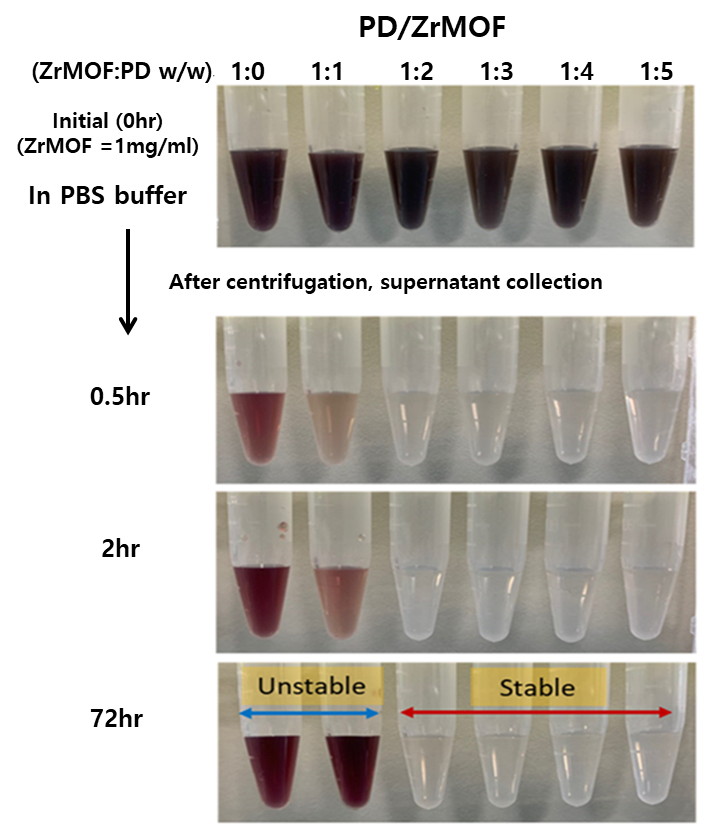


**Fig. S4** Stability test of the nanohybrids in PBS buffer (pH 7.4) at various ZrMOF:PD ratios. For the test, samples were centrifuged, and the supernatants were collected and visualized. Dark black supernatant suggests the degradation of MOFs.


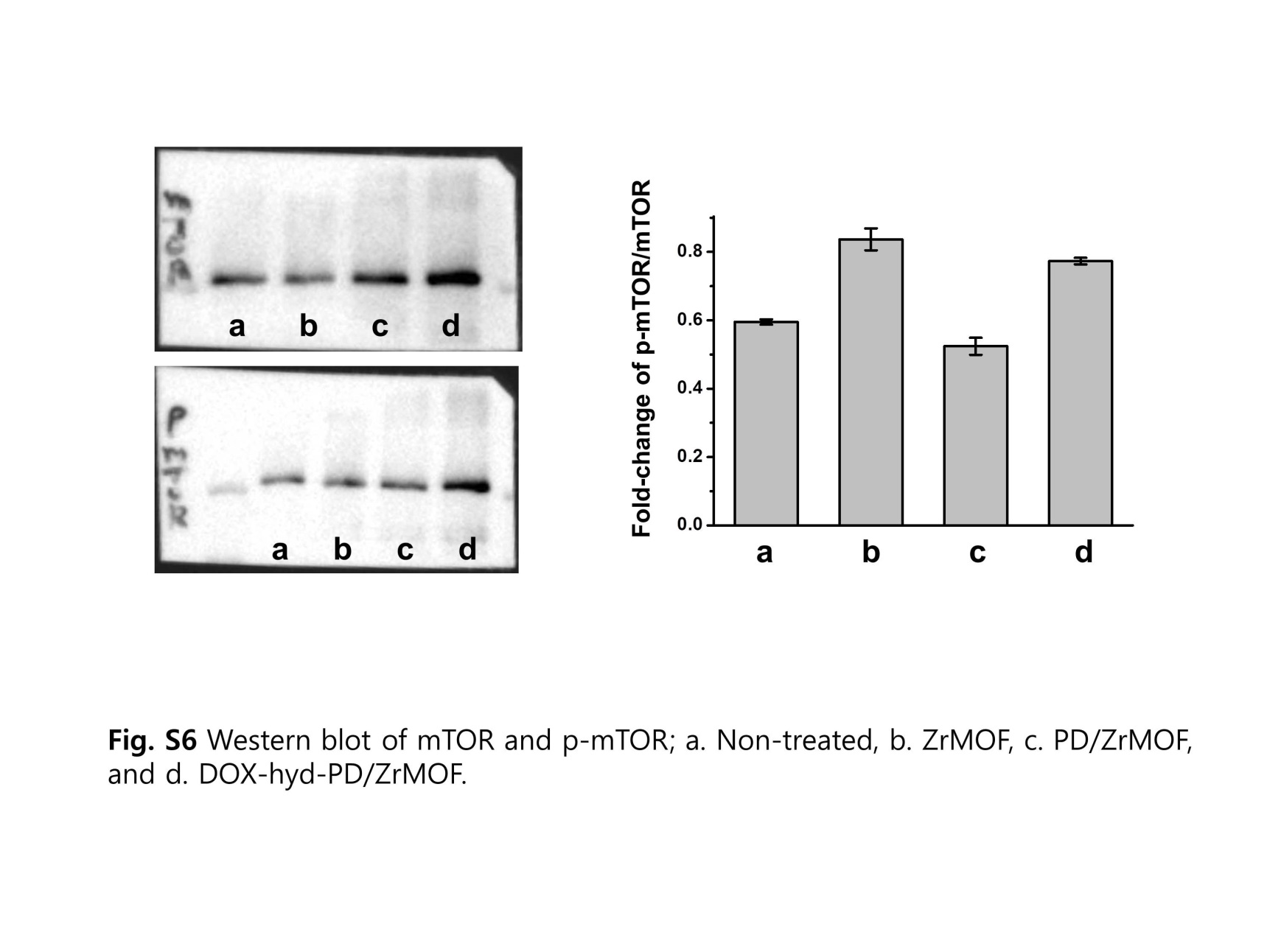


**Fig. S5** Western blot of mTOR and p-mTOR; a. Non-treated, b. ZrMOF, c. PD/ZrMOF, and d. DOX-hyd-PD/ZrMOF.


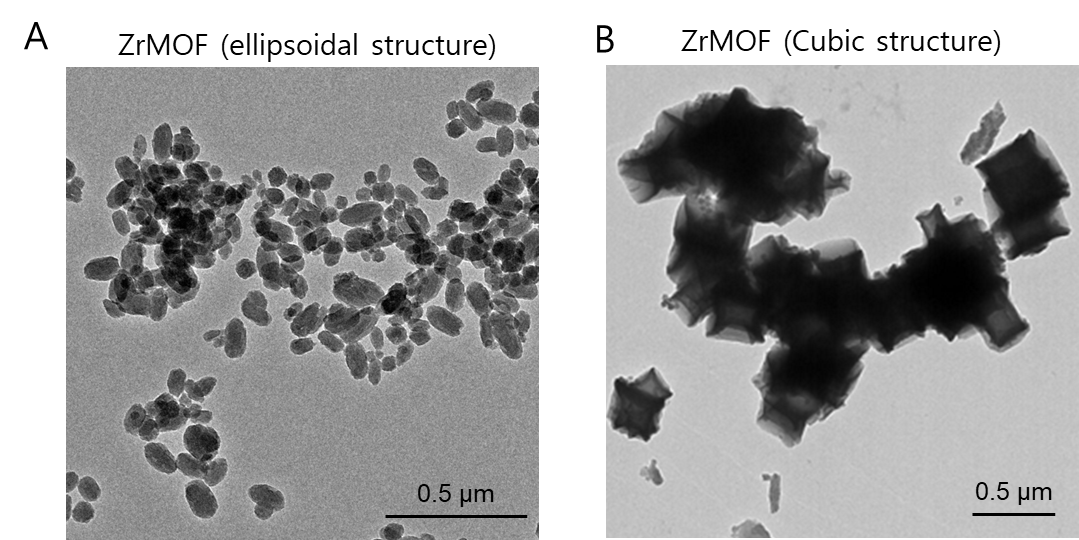


**Fig. S6** TEM images of ZrMOFs; (A) ellipsoidal structure, (B) Cubic structure
