# Supplementary figures and images for: Autophagy-Regulating, Photothermal Polydopamine-Coated, and Photodynamic Zirconium/Porphyrin-Framed Metal–Organic Frameworks for Enhanced Doxorubicin Therapy in Colon Cancer
Source: Biomater Res. 2025 Jun 12;29:0218. doi: 10.34133/bmr.0218 (PMC12160315; doi:10.34133/bmr.0218)

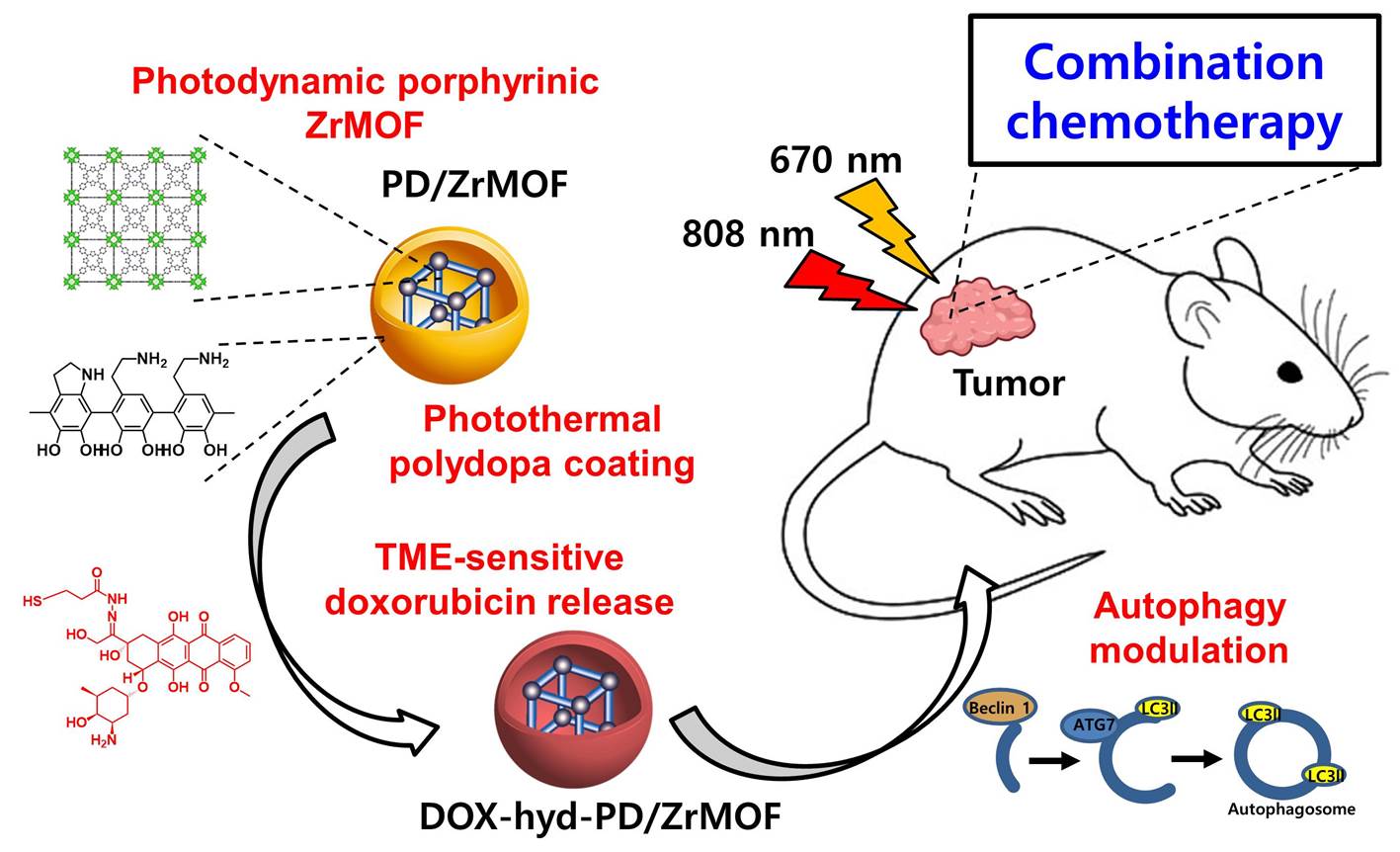

Supplement: Supplementary 1 — Graphical Abstract Figs. S1 to S6 [file bmr.0218.f1.zip › Abstract.jpg]
